# Supplementary material for: Comparison of microbial community structures in soils with woody organic amendments and soils with traditional local organic amendments in Ningxia of Northern China
Source: PeerJ. 2019 May 8;7:e6854. doi: 10.7717/peerj.6854 (PMC6511227; doi:10.7717/peerj.6854)
Supplement: Table S1 [file peerj-07-6854-s001.docx]

**Table S1** Chemical properties of soils and organic materials in this study

| Materirals | pH | Organic C (g kg^-1^) | Total N (g kg^-1^) | Total P (g kg^-1^) | Total K (g kg^-1^) |
| --- | --- | --- | --- | --- | --- |
| Soils | 8.74 | 0.54 | 0.13 | 0.15 | 15.44 |
| Cow manure | 8.58 | 201.25 | 13.5 | 3.70 | – |
| Corn straw | 6.67 | 450.50 | 6.51 | 1.44 | – |
| Poplar banches | 5.65 | 535.00 | 3.65 | 0.45 | – |

– not destermined.
