# Supplementary material for: Comparison of microbial community structures in soils with woody organic amendments and soils with traditional local organic amendments in Ningxia of Northern China
Source: PeerJ. 2019 May 8;7:e6854. doi: 10.7717/peerj.6854 (PMC6511227; doi:10.7717/peerj.6854)
Supplement: Table S2 [file peerj-07-6854-s002.docx]

**Table S2** Chemical properties of desertified soil with different organic amendments materials (means ± SE) **^†^**

| Time | Treatments | pH | SOC  (g kg^-1^) | TN  (g kg^-1^) | TP  (g kg^-1^) | TK  (g kg^-1^) | AN  (mg kg^-1^) | AP  (mg kg^-1^) | AK  (mg kg^-1^) | MBC  (mg kg^-1^) | MBN  (mg kg^-1^) |
| --- | --- | --- | --- | --- | --- | --- | --- | --- | --- | --- | --- |
| 7  months | Control | 8.64±0.14b | 1.31±0.11d | 0.15±0.01c | 0.23±0.01b | 15.75±0.30a | 66.00±13.77b | 15.04±3.41cd | 48.75±2.39e | 23.79±0.84d | 9.06±0.68c |
|  | CM | 8.63±0.09b | 2.04±0.08abc | 0.28±0.01a | 0.26±0.01ab | 16.43±0.24a | 102.25±6.14a | 64.98±4.32a | 467.50±21.36a | 16.06±0.85d | 13.59±0.60b |
|  | CS | 8.96±0.08ab | 1.88±0.08bc | 0.21±0.01b | 0.22±0.01b | 16.03±0.07a | 20.25±2.50d | 8.22±0.69d | 251.25±14.22c | 91.09±5.15b | 20.38±0.52a |
|  | PB | 9.00±0.06ab | 2.03±0.13abc | 0.22±0.01b | 0.22±0.00b | 16.10±0.11a | 29.25±2.46cd | 9.85±1.50d | 104.50±7.49e | 79.00±4.18b | 19.07±0.94a |
| 15  months | Control | 8.27±0.03a | 1.48±0.14b | 0.13±0.01c | 0.24±0.01bcd | 16.18±0.48a | 12.25±0.48c | 5.74±0.68d | 187.50±11.12b | 3.12±0.14d | 0.77±0.04e |
|  | CM | 7.89±0.09b | 2.10±0.14a | 0.27±0.01a | 0.31±0.01a | 16.85±3.77a | 76.25±3.77a | 67.15±3.08a | 448.75±9.66a | 10.69±0.47c | 2.15±0.12cd |
|  | CS | 7.91±0.04b | 2.23±0.07a | 0.24±0.01ab | 0.21±0.01d | 16.80±0.75a | 14.75±0.75c | 3.64±0.61d | 227.50±17.38ab | 23.47±1.93b | 3.20±0.22bc |
|  | PB | 8.02±0.03b | 2.25±0.04a | 0.23±0.00b | 0.22±0.01cd | 16.58±1.11a | 16.25±1.11c | 6.81±0.97d | 337.50±50.9ab | 18.04±1.31b | 2.50±0.20cd |

CS = corn straw, CM = cow manure, PB = poplar branch. Means with different letters are significantly different with *p* < 0.05 assessed by Tukey’s HSD test.
