# Supplementary material for: Comparison of microbial community structures in soils with woody organic amendments and soils with traditional local organic amendments in Ningxia of Northern China
Source: PeerJ. 2019 May 8;7:e6854. doi: 10.7717/peerj.6854 (PMC6511227; doi:10.7717/peerj.6854)
Supplement: Table S3 [file peerj-07-6854-s003.docx]

**Table S3** Effects of each treatment on distributions of microbial phyla

| Time | Treatment | Bacterial | | |  | Fungal | | |
| --- | --- | --- | --- | --- | --- | --- | --- | --- |
|  |  | df | *F* | *p* |  | df | *F* | *p* |
| 7 months | Control | 8 | 8.42 | < 0.001 |  | 4 | 9.088 | 0.001 |
|  | CM | 8 | 11.309 | < 0.001 |  | 4 | 48.909 | < 0.001 |
|  | CS | 8 | 43.48 | < 0.001 |  | 4 | 29.519 | < 0.001 |
|  | PB | 8 | 29.517 | < 0.001 |  | 4 | 36.802 | < 0.001 |
| 15 months | Control | 8 | 17.17 | < 0.001 |  | 4 | 165.073 | < 0.001 |
|  | CM | 8 | 37.692 | < 0.001 |  | 4 | 149.099 | < 0.001 |
|  | CS | 8 | 601.383 | < 0.001 |  | 4 | 5097.389 | < 0.001 |
|  | PB | 8 | 177.177 | < 0.001 |  | 4 | 79.553 | < 0.001 |

Values represent the pseudo-F ratio (*F*) and the level of significance (*p*).
